# Undifferentiated Pleomorphic Sarcoma of Liver: Case Report and Review of the Literature

**DOI:** 10.1155/2018/8031253

**Published:** 2018-07-17

**Authors:** Jirong (Betty) Mass, Geoffrey Talmon

**Affiliations:** University of Nebraska Medical Center, Omaha, NE, USA

## Abstract

Undifferentiated pleomorphic sarcoma (UPS), previously known as malignant fibrous histiocytoma (MFH), is rarely reported in the liver as a primary site. We report a case of a previously healthy 56-year-old male, who presented with abdominal pain and jaundice. The patient was originally diagnosed with cholecystitis, treated with cholecystectomy, which was complicated by abdominal abscess. One week following discharge, the patient was readmitted with fever, chills, and leukocytosis. Computed tomography (CT) guided liver biopsies demonstrated an epithelioid to spindle cell neoplasm with markedly atypical nuclei and prominent necrosis infiltrating between hepatocytes. Immunohistochemical studies were negative for epithelial, melanocytic, and hematolymphoid differentiation. Positron emission tomography (PET) was performed, which showed a single markedly hypermetabolic central hepatic mass (14 x 8.5 x 8.5 cm) with likely central necrosis, consistent with primary malignancy. The patient was treated with one cycle of chemotherapy (doxorubicin and ifosfamide), refusing additional cycle due to medication side effects. The patient subsequently succumbed to complications associated with the malignancy and died within 19 days of diagnosis.

## 1. Introduction

Undifferentiated pleomorphic sarcoma (UPS), previously known as malignant fibrous histiocytoma (MFH), was first reported in 1964 by O'Brien and Stout [1, 2]. It has been widely recognized as one of the most common malignant soft tissue tumors usually occurring late in adult life [3, 4]. UPS typically involves the extremities and less commonly the retroperitoneal spaces, abdominal cavity, or other sites such as visceral organs [5].

The first case of primary hepatic UPS was described in 1985 [6]. Fewer than 200 cases have been reported [7] and our understanding of the tumor is still very limited. In this article, a case of primary hepatic UPS treated in our hospital is reported and the recent literature of UPS is reviewed.

## 2. Case Presentation

A previously healthy 56-year-old man suffering from abdominal pain and jaundice was admitted with an initial clinical diagnosis of acute cholecystitis. The patient underwent an endoscopic retrograde cholangiopancreatogram (ERCP) and cholecystectomy. His symptoms did not improve and repeat imaging study indicated common bile duct narrowing. A 20 x 3.5 cm perihepatic abscess was found that required drainage and he underwent percutaneous transhepatic cholangiography (PTC) and biliary drainage. The cytologic examination was not performed on the drained material. Laboratory studies at that time revealed the following: WBC: 51.4 x 10^9^/L (N: 4.5-11.0 x 10^9^/L), Hb: 9.9 g/dL (N: 13.5-17.5 g/dL), serum Na^+^ 129 mEq/L (N: 135-145 mEq/L), serum K^+^ 3.4 mEq/L (3.5-5.0 mEq/L), serum albumin: 2.1 g/dL (N: 3.5-5.0 g/dL), lipase 303 U/L (N: 0-50 U/L), and AST/ALT 93/97 U/L (N: AST/ALT: 8-20/8-20 U/L). The patient was discharged on antibiotics after three weeks of treatment. One week later, he developed a fever, chills, and leukocytosis. He was readmitted into hospital. Abdominal CT showed multiple fluid collections within the liver parenchyma with the largest one being 2.2 x 2.0 cm in size. A CT guided liver biopsy of the presumed abscess was performed.

**Figure 1:**
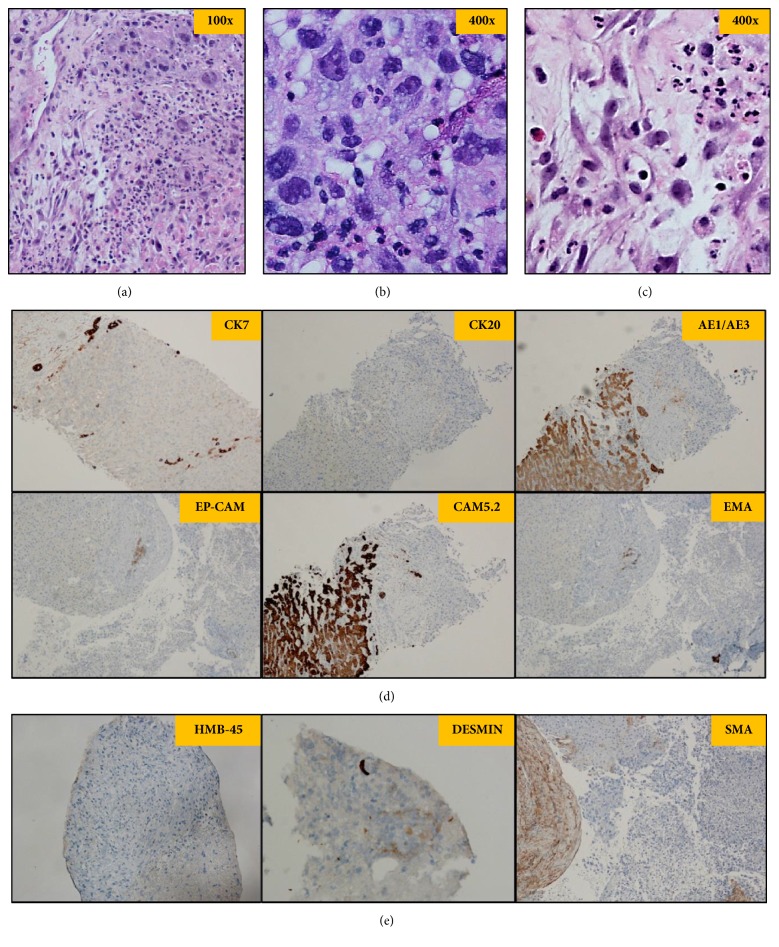
The biopsy showed the tumor was composed of epithelioid to spindle cells infiltrating between hepatocytes with markedly atypical nuclei and prominent necrosis, exhibiting a pleomorphic pattern. (a) Hematoxylin and eosin stain at 100x magnification; (b) and (c) hematoxylin and eosin stain at 400x magnification; (d) Epithelial-Panel staining; (e) other linage staining: HMB-45, desmin, and SMA (the rest of staining not shown).

The biopsy showed an epithelioid to spindle cell neoplasm infiltrating between hepatocytes with markedly atypical nuclei and prominent necrosis (Figures 1(a), 1(b), and 1(c)). The tumor exhibited a pleomorphic pattern. Extensive immunostaining was performed, including hepatocellular carcinoma markers (AFP, HepPar1, Glypican-3, polyclonal CEA, and ARG1), other epithelial antigens (CK7, CK20, AE1/AE3, CAM5.2, EpCAM, and EMA) (Figure 1(d)), Inhibin, CD117, CD30, and CD3, and ALK-monoclonal, germ cell markers (AFP, OCT3/4, and HCG), melanoma markers (Melan-A, S-100, and SOX10), and endothelial (CD31) and muscle (smooth muscle actin) markers (Figure 1(e)) were all negative. The tissue was exhausted.

Based on the inconclusive findings, a second liver biopsy was performed. The morphology was similar to the prior biopsy. Further staining was performed. The tumor cells were also negative for HMB-45, CD15, CD20, CD21, CD23, CD43, CD45, desmin, myogenin, calretinin, myeloperoxidase, D2-40, CD68, and clusterin (Figure 1(e)). Based on the radiographic features in combination with the morphology and immunophenotype, it was likely a primary hepatic lesion without epithelial, melanocytic, or hematolymphoid differentiation. As such, a primary liver sarcoma was favored.

**Figure 2:**
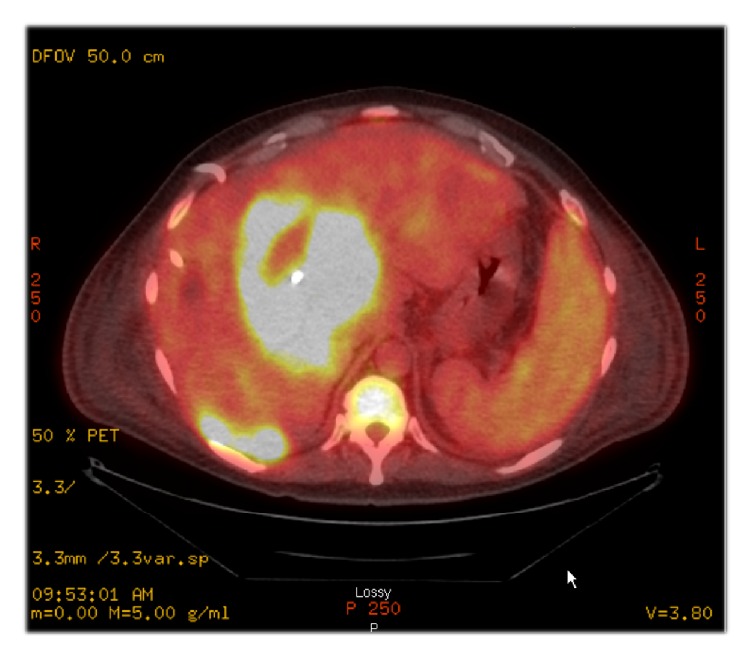
Postbiopsy PET/CT result.

Following the biopsies, the physician in charge ordered a PET/CT after reviewing the biopsy results in order to evaluate tumor size and potential metastasis (Figure 2). A large markedly hypermetabolic central hepatic mass (14.0 x 8.5 x 8.5 cm) with likely central necrosis was identified, consistent with primary malignancy. Additionally, there were multifocal hypermetabolic liver lesions and hypermetabolic peritoneal implants suggesting peritoneal dissemination.

The patient was treated with one cycle of chemotherapy (adriamycin and ifosfamide) which caused severe confusion and further treatment was refused. The patient expired within 19 days of diagnosis.

## 3. Discussion

UPS refers to a group of pleomorphic sarcomas that lack any specific line of differentiation [5]. In fact, the reason for the disuse of the old name of the entity—“malignant fibrous histiocytoma”—was a reflection of this definition: UPS does not demonstrate evidence of specific mesenchymal cell differentiation [2].

Primary hepatic UPS is a tumor of late adulthood with a mean age of 58 [8]. No sex predisposition has been described [8]. Some cases are associated with radiation exposure with other cases not having a strong etiologic link [5, 9]. Symptoms are usually nonspecific including weight loss, anorexia, fever, jaundice, malaise, right upper quadrant pain, and palpable abdominal mass [8]. Unremarkable laboratory results are commonly seen [8].

Grossly, UPS is often white to pale yellow, with central hemorrhage and necrosis [5]. The tumor affects all portions of the liver, with an average size of at least 12 cm [8]. It is histologically characterized by high cellularity, marked nuclear pleomorphism, abundant mitotic activity (including atypical mitoses), and areas with a spindle cell morphology [5]. Necrosis is a common feature of high grade lesions [5]. Immunostaining is often not revealing. Although histiocytic markers have no role in its diagnosis simply because this tumor does not display true histiocytic differentiation, UPS cells often express CD68. However, CD68 may be interpreted as positive due to the relatively high number of tumor infiltrating histiocytes in UPS [10]. As such, UPS is a diagnosis of exclusion [5]. Particularly, sarcomatoid carcinomas, leiomyosarcomas, epithelioid PEComas must be ruled out. The histologic differential diagnosis also included undifferentiated (embryonal) sarcoma (which is less likely given the patient's age, lack of biphasic morphology, and antecedent liver mass) and histiocytic dendritic neoplasm. Malignant mesothelioma could be one of the differential diagnoses as well. The negativity of calretinin in this case can help us to rule it out. Recent therapies including the remedy targeting programmed death 1 (PD-1) or its ligand (PD-L1) represent novel insights of the immunotherapy of the treatment of UPS [11]. However, the only established treatment for UPS is surgery, with or without radiotherapy. After treatment, local recurrence rates range from 19 to 31%, with a metastatic rate of 31–35% and a 5-year survival rate of 65–70% [5].

Clinically, in the present case, possible peritoneal dissemination was noted and was suggested by the peritoneal implants in the PET image (Figure 2). Commonly, the lesions of UPS were solitary and the metastases to regional lymph node were rare. To investigate if peritoneal dissemination was a common finding to hepatic UPS, a review of the previous literature was performed. In one previous study, seventeen percent (13/76) of hepatic UPS were reported to have multiple lesions involved but none of them was reported to have peritoneal dissemination at the time of diagnosis [7]. There was one case in the same series that the patient was found to have intraperitoneal plant following the drainage of the cystic UPS which was originally misdiagnosed as a liver cyst [7]. However, there was no peritoneal dissemination reported by the time he was diagnosed [7]. Eight cases were reported to have direct invasion of the adjacent organs [8, 12, 13] but no peritoneal dissemination was identified at diagnoses in those cases. Therefore, the peritoneal dissemination may not be considered as a common feature of hepatic UPS. The peritoneal dissemination in the current case might be explained by the previous drainage of the previous abscess.

For the origin of UPS cells, many scientists and pathologists believe the “dedifferentiation theory.” In this model it is presumed that varying tumors with shared similar morphologic features become progressively more undifferentiated, ultimately resulting in a high grade undifferentiated pleomorphic sarcoma [14–16]. Others have postulated that UPS is actually the results of transformation of mesenchymal stem cells (MSC) [14–16].

Several studies suggested that UPS and pleomorphic leiomyosarcoma potentially share a linage due to significant similarities between them [17, 18]. Böhling's study reported CGH results from 102 MFH and 82 LMS cases, as well as a subsequent clustering analysis. There was no significant difference of the distribution pattern of DNA copy number between LMS and MFH, suggesting that most MFHs could represent a final state of tumor progression of LMS. Their data, however, also suggested that even if an oncogenic pattern common to LMS and MFH was demonstrated, the genes associated with smooth muscle cell differentiation may locate in one or more chromosomal imbalances that are not shared by both tumor types [17].

Researchers also compared UPS with myogenic differentiation (MD) and non-MD UPS in soft tissue hypothesizing that UPS with MD would be more aggressive than non-MD UPS as pleomorphic sarcoma would suggest. However, no survival rate difference was demonstrated between them [19].

In the past several decades since UPS was firstly recognized, tremendous effort has been directed towards reclassification of UPS based on clinical behavior due to its significance for the treatment. Multiple strategies have been applied in recent years including comparative genomic hybridization (CGH) analysis [20], cDNA microarray [21], and proteomics analysis [2, 22, 23].

Meanwhile, a number of recurrent chromosomal regions of gain and loss have been identified in soft tissue UPS [24]. Some of them were reportedly associated with better patient survival [9, 24]. Another study [25] showed that polysomic chromosomes appeared more characteristically in UPS; however, in their study sarcoma-specific chromosomal breaks and oncogene amplifications were rarely identified. There was also an investigation that indicated loss of 4q31 (encompassing the SMAD1 gene) and loss of 18q22 as independent predictors of metastasis [5].

Recently, a study of a prognostic miRNA biomarker (miRNA138) for clinical validation was identified along with a RHO-ROCK cell adhesion pathway that modulates the UPS metastatic phenotype [26] which provide another possible explanation of UPS metastatic mechanism and a potential clinical prognostic marker. Interestingly, another group reported RAS/MAPK and PI3K/mTOR pathways were activated in the majority (> 80%) of cases of UPS in their study [27, 28]. It suggested that the activation of RAS/MAPK pathway distinguished a subgroup of patients with localized UPS with a worse outcome [27, 28]. Yes-associated protein 1 (YAP1) was also reported to play a role in molecular mechanism in UPS and demonstrate the potential impact to the treatment [29].

In sum, although hepatic UPS is a rare malignant mesenchymal tumor with a nonspecific clinical and radiologic presentation, it should be considered in the diagnosis of large liver lesions without evidence of differentiation. Surgical resection is the most effective means for treating this rare tumor and the prognosis usually is poor. Recent progress in UPS research has allowed for a potential classification system and potential therapeutic targets [14, 21, 22, 30–32].
